# Neural predisposing factors of postoperative delirium in elderly patients with femoral neck fracture

**DOI:** 10.1038/s41598-018-26030-2

**Published:** 2018-05-15

**Authors:** Sunghyon Kyeong, Jung Eun Shin, Kyu Hyun Yang, Woo Suk Lee, Tae-Sub Chung, Jae-Jin Kim

**Affiliations:** 1 Institute of Behavioral Sciences in Medicine, Yonsei University College of Medicine, Seoul, Republic of Korea; 2 Department of Orthopedic Surgery, Yonsei University College of Medicine, Seoul, Republic of Korea; 3 Department of Radiology, Yonsei University College of Medicine, Seoul, Republic of Korea; 4 Department of Psychiatry, Yonsei University College of Medicine, Seoul, Republic of Korea

## Abstract

Elderly adults are more likely to develop delirium after major surgery, but there is limited knowledge of the vulnerability for postoperative delirium. In this study, we aimed to identify neural predisposing factors for postoperative delirium and develop a prediction model for estimating an individual’s probability of postoperative delirium. Among 57 elderly participants with femoral neck fracture, 25 patients developed postoperative delirium and 32 patients did not. We preoperatively obtained data for clinical assessments, anatomical MRI, and resting-state functional MRI. Then we evaluated gray matter (GM) density, fractional anisotropy, and the amplitude of low-frequency fluctuation (ALFF), and conducted a group-level inference. The prediction models were developed to estimate an individual’s probability using logistic regression. The group-level analysis revealed that neuroticism score, ALFF in the dorsolateral prefrontal cortex, and GM density in the caudate/suprachiasmatic nucleus were predisposing factors. The prediction model with these factors showed a correct classification rate of 86% using a leave-one-out cross-validation. The predicted probability computed from the logistic model was significantly correlated with delirium severity. These results suggest that the three components are the most important predisposing factors for postoperative delirium, and our prediction model may reflect the core pathophysiology in estimating the probability of postoperative delirium.

## Introduction

Delirium is an acute neuropsychiatric syndrome that is characterized by sudden alterations and fluctuations in consciousness and cognition. The risk factors for delirium include aging, cognitive and sensory impairment, and major surgery^1,2^, and various clinical and psychological risk factors have been additionally identified^3–6^. In a general hospital, elderly patients are more likely to develop delirium, particularly after a major surgery. It is worth noting that delirium has a considerable impact on prognosis even though postoperative delirium mostly disappears within a few days. In fact, delirium can lead to adverse consequences including persistent cognitive decline^7,8^.

Although elderly adults who are the same age undergo the same operation, some develop postoperative delirium while others do not, suggesting the existence of neural predisposing factors and the need for creating a prediction model. Identification of the neural predisposing factors is also important to shed light on the pathology of delirium. The prediction of delirium may be useful because early intervention reduces the duration of delirium, length of hospitalization, and mortality in delirious patients^9^. Nonetheless, the lack of known neural predisposing factors and a clinically available prediction model appears somewhat surprising given that numerous studies have identified risk factors of delirium.

Because delirium is a neurocognitive disorder accompanying brain dysfunction, several studies have been conducted to identify the neural substrates of delirium. For example, structural neuroimaging studies have shown an association between abnormalities in the brain volume and clinical outcomes such as the duration of delirium and decreased cognition^10^. Our group reported that hyperconnectivity of functional networks between the posterior cingulate cortex (PCC) and dorsolateral prefrontal cortex (DLPFC) was observed during an episode of delirium^11^. Unfortunately, neurobiological evidence of delirium from these two studies could not be used to predict postoperative delirium because it was acquired during and after the resolution of delirium.

With the known risk factors for delirium, the prediction model has shown its usefulness in clinical practice. For example, some prediction model of delirium including ten risk factors such as demographic, clinical, and laboratory data was applied to patients in intensive care unit, and its accuracy was higher than that of clinical prediction by nurses and physicians^12^. The prediction model using the levels of cognitive decline, illness severity, and laboratory data has been proposed to predict postoperative delirium in elderly hip-surgery patients^13^. Some prospective cohort studies have shown the predictive model of delirium in hospitalized elderly patients using 4 risk factors such as vision impairment, severity illness, cognitive impairment, and blood urea nitrogen/creatinine ratio^14^. Despite various suggestions, these previous models have not included neurobiological evidence. Considering that the etiology of delirium is multifactorial, its risk factors are likely to interact in a complex way^15^. Therefore, the development of prediction model including various types of data such as clinical assessment, laboratory data, and neurobiological data would be necessary to improve its efficacy.

In the present study, we tried to identify the neural predisposing factors of postoperative delirium using assessments of the preoperative anatomical and resting-state functional magnetic resonance imaging (rsfMRI) data. Participants were preoperative patients over 70 years old with femoral neck fractures, since a higher rate of postoperative delirium has been reported in older patients in the orthopedic unit than in other surgical units^1^. Additionally, we believe that examining patients from a single unit is beneficial to reduce the influence of various undefined risk factors for postoperative delirium in the clinical field. Preliminary to the present study, we recently identified psychological risk factors of postoperative delirium including neuroticism^5^. According to this perspective, we only considered the cognitive and personality characteristics identified by our preliminary study rather than re-assessing all possible clinical features, and instead focused on identifying neural predisposing factors from structural and functional brain imaging data in the present study.

In addition, this study aimed to create a prediction model for estimating an individual’s probability of postoperative delirium. In order to determine the best prediction model, we considered three models with different types of features: a clinical model with assessments of personality and cognitive decline; a biological model with neuroimaging features obtained from the structural and functional MRI; and a combined model with clinical and biological features. We expected that the prediction models could estimate the severity of delirium.

## Results

### Characteristics of participants

**Figure 1:**
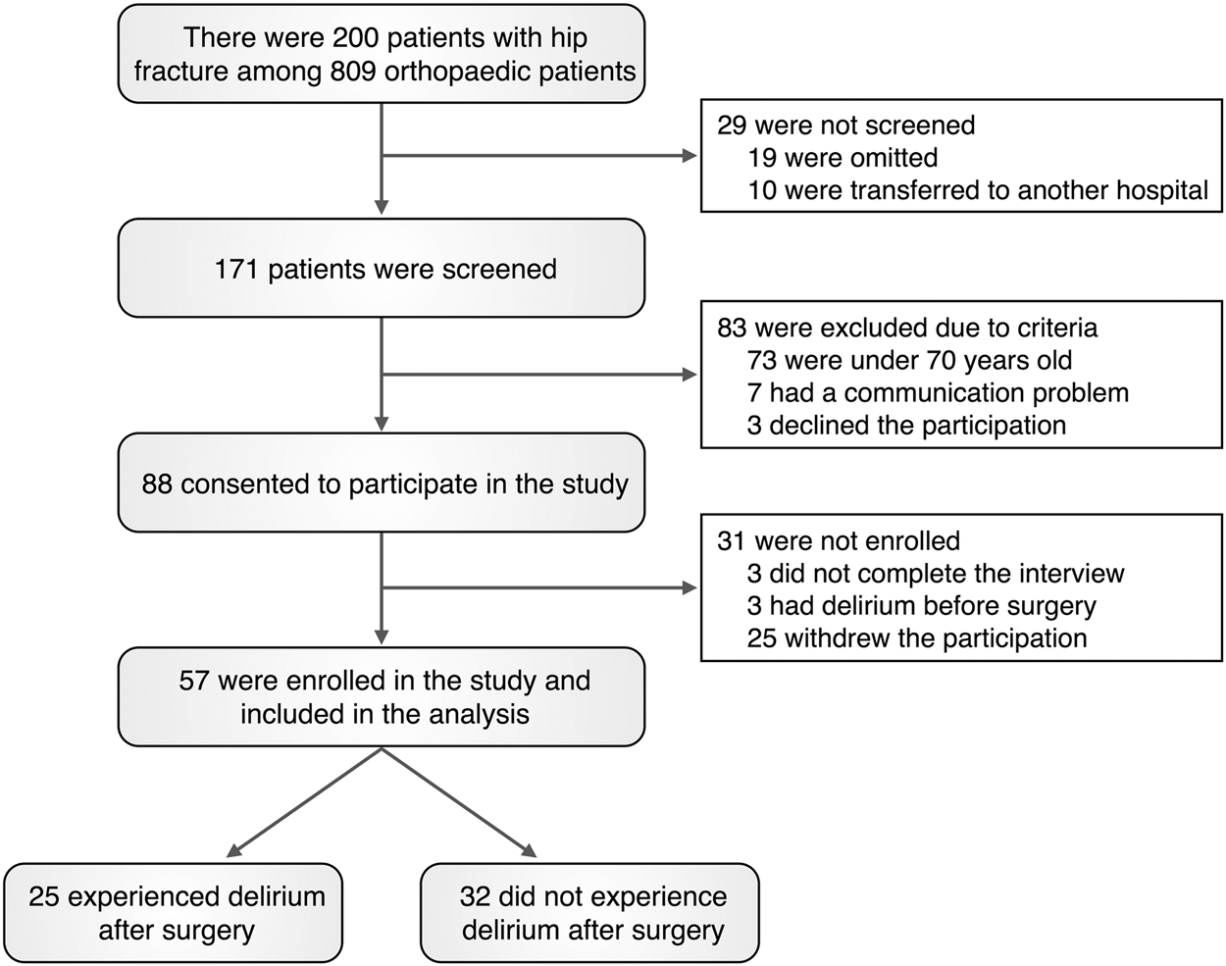
Flowchart for participant enrollment.

Among 809 orthopedic patients who were admitted during a consecutive 11-month study period to Yonsei University Gangnam Severance Hospital, a total of 57 elderly patients (≥70 years old) who fractured their femur neck due to falling were included in the current study (see Fig. 1 for detailed enrollment procedures). Among 57 patients, 25 participants developed postoperative delirium (DEL group), but remaining 32 participants did not develop postoperative delirium (No-DEL group). There were no statistical differences in age, gender, education duration, past medical history, and family history between the two groups (Table 1). The Mini-Mental State Examination (MMSE) scores were significantly lower in the DEL group than in the No-DEL group (*t* = −2.41, *p* = 0.02). Among the five dimensions of the Big Five Inventory (BFI), neuroticism was the only dimension that showed significantly higher scores in the DEL group than in the No-DEL group (*t* = 3.61, *p* < 0.01). The DEL group mostly showed delirium symptoms on postoperative day 2 (range: 1–5), which lasted for 4 days (range: 1–33). The mean total score of the Korean version of the Delirium Rating Scale-Revised-98 (KDRS) was 19.8 (range: 12–28) (see Table 2 for details).

**Table 1:** Characteristics of participants according to postoperative delirium.

Variable	DEL group (*n* = 25)	No-DEL group (*n* = 32)	*T* or χ²	*P*
Demographics				
Age, mean (SD), year	83.7 (6.1)	80.9 (6.6)	1.64	0.11
Female sex, No. (%)^†^	18 (72%)	29 (91%)	3.37	0.07
Education, mean (SD), year	8.7 (4.2)	7.9 (4.9)	0.64	0.53
Medical history, No. (%)^†^				
Major mental disorder history	5 (20%)	3 (9%)	1.31	0.28
Dementia history	5 (20%)	3 (9%)	1.31	0.28
Delirium history	4 (16%)	2 (6%)	1.22	0.39
Other mental disorder history	1 (4%)	1 (3%)	0.32	1.00
Brain injury history	3 (12%)	6 (19%)	0.56	0.72
Psychiatry family history	8 (32%)	6 (19%)	1.33	0.25
Hypertension	18 (72%)	22 (69%)	0.07	0.79
Diabetes	8 (32%)	10 (31%)	0.00	0.95
Cognitive measure, mean (SD)				
MMSE, score	16.5 (6.3)	20.5 (6.2)	−2.41	0.02
Personality scale, mean (SD)				
BFI-Agreeableness, score	6.2 (2.4)	6.8 (1.5)	−1.19	0.24
BFI-Conscientiousness, score	7.8 (1.9)	8.2 (1.8)	−0.91	0.37
BFI-Extraversion, score	6.2 (1.9)	6.3 (1.4)	−0.21	0.84
BFI-Neuroticism, score	7.7 (1.3)	6.5 (1.2)	3.61	0.00
BFI-Openness, score	6.1 (2.1)	6.5 (1.3)	−0.87	0.39

Abbreviations: BFI, Big-Five Inventory; DEL group, participants who developed postoperative delirium; MMSE, Mini-Mental State Examination; No, number; No-DEL group, participants who did not develop postoperative delirium; SD, standard deviation.

^†^Fisher test were applied to examine group differences.

**Table 2:** Summary of Delirium Rating Scale in delirious patients. Mean and standard deviation (SD) for each rating score are presented.

Delirium Rating Scale		Mean ± SD
**Severity items**		**19.8 ± 4.4**
item1	Sleep-wake cycle disturbance	1.6 ± 0.8
item2	Perceptual disturbances and hallucinations	1.3 ± 1.3
item3	Delusions	0.0 ± 0.0
item4	Lability of affect	1.0 ± 0.6
item5	Language	1.4 ± 0.8
item6	Thought process abnormalities	1.8 ± 1.0
item7	Motor agitation	0.8 ± 0.9
item8	Motor retardation	1.4 ± 0.9
item9	Orientation	2.1 ± 0.8
item10	Attention	2.0 ± 0.6
item11	Short-term memory	2.0 ± 1.3
item12	Long-term memory	1.7 ± 1.2
item13	Visuospatial ability	2.7 ± 0.7
**Diagnostic items**		6.0 ± 0.2
item14	Temporal onset of symptoms	3.0 ± 0.0
item15	Fluctuation of symptom severity	**1.0 ± 0.2**
item16	Physical disorder	2.0 ± 0.0

### Group Differences in Neuroimaging Variables

**Figure 2:**
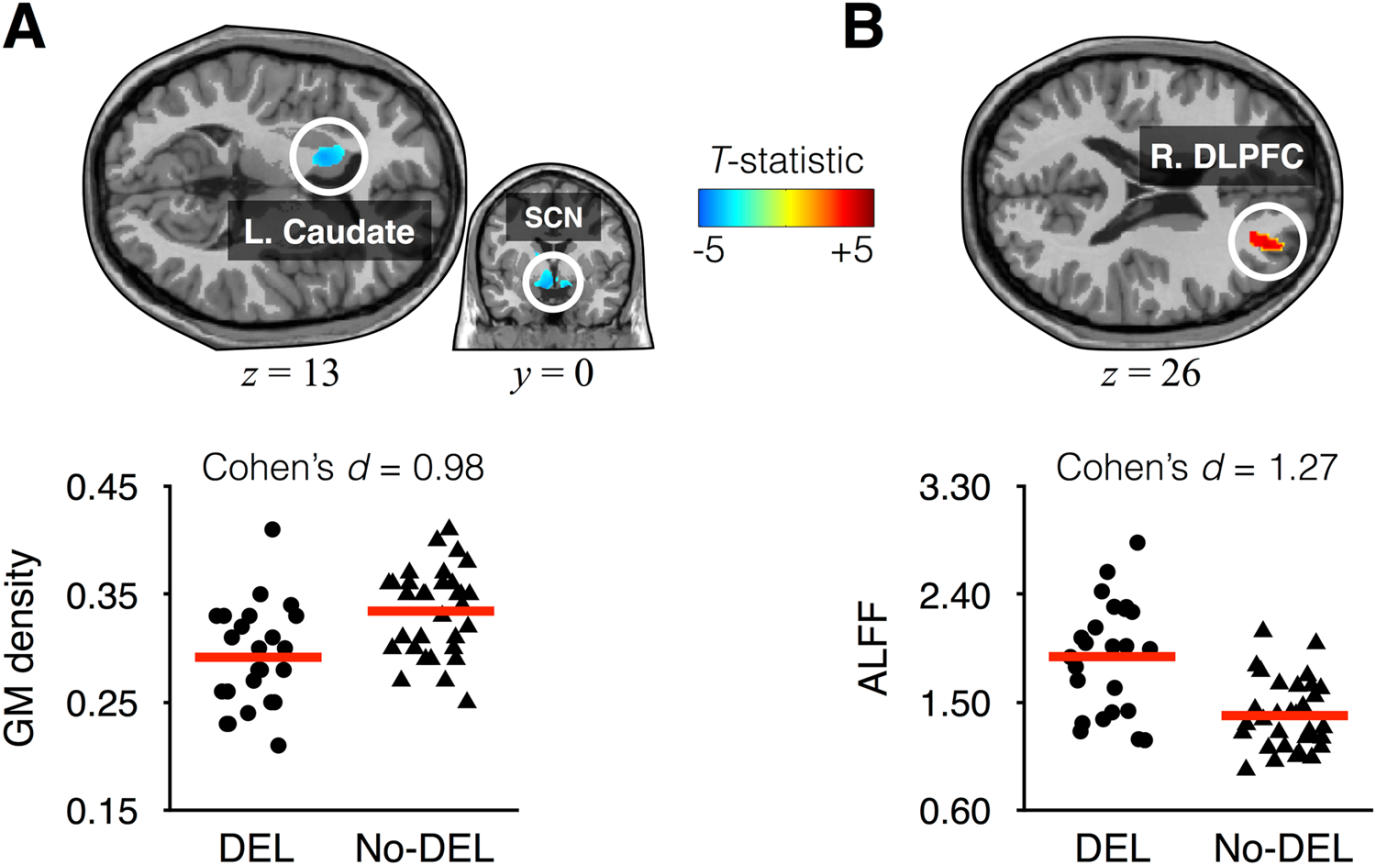
Mapping brain regions playing essential roles in prediction models. Gray matter (GM) density in the left caudate and suprachiasmatic nucleus (SCN) was significantly d00000000000ecreased in the delirium (DEL) group relative to the non-delirium (No-DEL) group (**A**), whereas the amplitude of low-frequency fluctuation (ALFF) in the right dorsolateral prefrontal cortex (R. DLPFC) was significantly increased in the DEL group relative to the No-DEL group (**B**).

Voxel-based morphometry revealed that the DEL group had structural atrophy in multiple brain regions (Table 3). Relative to the No-DEL group, the DEL group showed decreased gray matter (GM) density in the right dorsomedial prefrontal cortex/supplementary motor area, left paracentral lobule, left middle temporal gyrus, right inferior temporal gyrus, left caudate/suprachiasmatic nucleus (corrected *P* < 0.05, Cohen’s *d* >0.8), right PCC, left hippocampus, and left cerebellum (corrected *P* < 0.05, 0.5< Cohen’s *d* <0.8). The DEL group showed decreased fractional anisotropy (FA) in the right external capsule compared with the No-DEL group (corrected *P* < 0.05, Cohen’s *d* = 0.82). The amplitude of low frequency fluctuation (ALFF) in the right DLPFC computed from the preoperative rsfMRI data was significantly increased in the DEL group compared with the No-DEL group (corrected *P* < 0.05, Cohen’s *d* = 1.27). Among the multiple brain regions showing significant group differences, Fig. 2 shows the most important structural and functional properties that played a pivotal role in the prediction model as described in the following section. Meanwhile, the framewise displacement representing head movements during rsfMRI scans did not significantly differ between the two groups (*t*_54_ = 1.8, *P* = 0.08).

**Table 3:** Summary of brain regions showing significant differences between the DEL and No-DEL groups in gray matter density, fractional anisotropy, and amplitude of low-frequency fluctuation.

Region	DEL group	No-DEL group	MNI coordinates	Volume	Zmax	Cohen’s *d*
	Mean (SD)	Mean (SD)	*x, y, z*	cc		
**Gray matter density**						
DEL > No-DEL						
No significant results						
DEL < No-DEL						
R. DMPFC/pre-SMA	0.376 (0.048)	0.422 (0.067)	8, 20, 53	4.51	4.48	0.81
R. PCC	0.383 (0.051)	0.422 (0.055)	10, −34, 39	2.47	4.73	0.75
L. Paracentral lobule	0.306 (0.047)	0.349 (0.061)	−2, −23, 67	2.36	4.47	0.82
L. Middle temporal gyrus	0.391 (0.065)	0.441 (0.052)	−55, −23, −16	11.27	5.59	0.81
R. Inferior temporal gyrus	0.395 (0.052)	0.442 (0.058)	48, 5, −36	9.50	5.24	0.82
L. Hippocampus	0.358 (0.069)	0.397 (0.057)	−37, −19, −17	1.88	4.58	0.57
L. Caudate	0.292 (0.046)	0.335 (0.040)	−14, 9, 13	4.79	5.42	0.98
L. Suprachiasmatic nucleus			−8, 0, −16		4.61	
R. Suprachiasmatic nucleus			11, −1, −15		4.58	
L. Cerebellum	0.433 (0.048)	0.478 (0.083)	−42, −71, −54	2.43	4.31	0.61
L. Cerebellum	0.483 (0.056)	0.525 (0.068)	−23, −44, −29	2.65	3.89	0.63
R. Cerebellum	0.431 (0.056)	0.459 (0.053)	20, −41, −29	2.84	3.93	0.48
**Fractional anisotropy**						
DEL > No-DEL						
No significant results						
DEL < No-DEL						
R. External capsule	0.218 (0.035)	0.244 (0.028)	33, 14, −3	2.35	4.76	0.82
**Amplitude of low-frequency fluctuation**						
DEL > No-DEL						
R. DLPFC	1.880 (0.462)	1.389 (0.291)	24, 38, 26	1.31	4.90	1.27
DEL < No-DEL						
No significant results						

Abbreviations: cc, cubic centimeters; DEL group, participants who developed postoperative delirium; DLPFC, dorsolateral prefrontal cortex; DMPFC, dorsomedial prefrontal cortex; SD, standard deviation; MNI, Montreal Neurological Institute; No-DEL group, participants who did not develop postoperative delirium; SMA, supplementary motor area; Zmax, maximum z-value within a cluster.

### Relationships between Neuroimaging Results and Symptom Severity

In partial correlation analysis within the DEL group, KDRS severity total scores were negatively correlated with FA value in the external capsule (*r* = −0.44, Corrected *P* = 0.04). Furthermore, the severity of perceptual disturbances and hallucinations (item 2 of the KDRS) was significantly correlated with GM density in the middle temporal gyrus (*r* = 0.67, Corrected *P* = 0.01), hippocampus (*r* = 0.58, Corrected *P* = 0.02), and caudate/suprachiasmatic nucleus (*r* = 0.61, *P* = 0.02), and with FA value in the external capsule (*r* = 0.54, Corrected *P* = 0.01). Except for this item, no significant correlations were observed between other item scores and neuroimaging results.

### Group Differences in Small-world Network Properties

**Figure 3:**
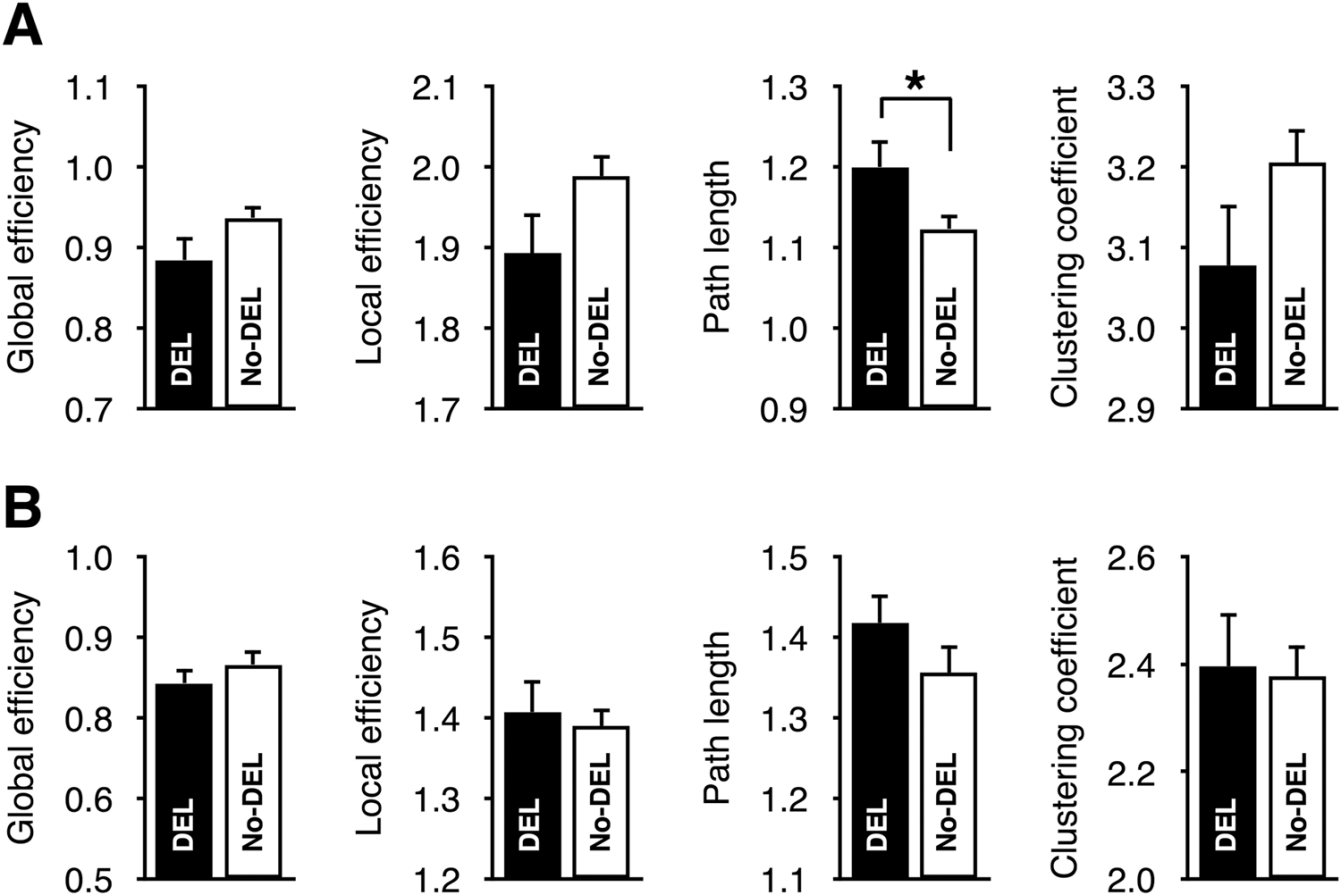
Small-world network properties of the structural (**A**) and functional (**B**) networks. All measures were normalized by that of 1,000 random networks. Abbreviation: DEL, delirium; No-DEL, non-delirium.

Fig. 3 shows the small-world properties of the structural and functional networks. Relative to the No-DEL group, only the characteristic path length of the structural network was significantly increased in the DEL group (*P* = 0.03, Cohen’s *d* = 0.57). No significant group differences were observed in the small-world properties of the functional network.

### Variables Remaining Significant in Each Model

**Figure 4:**
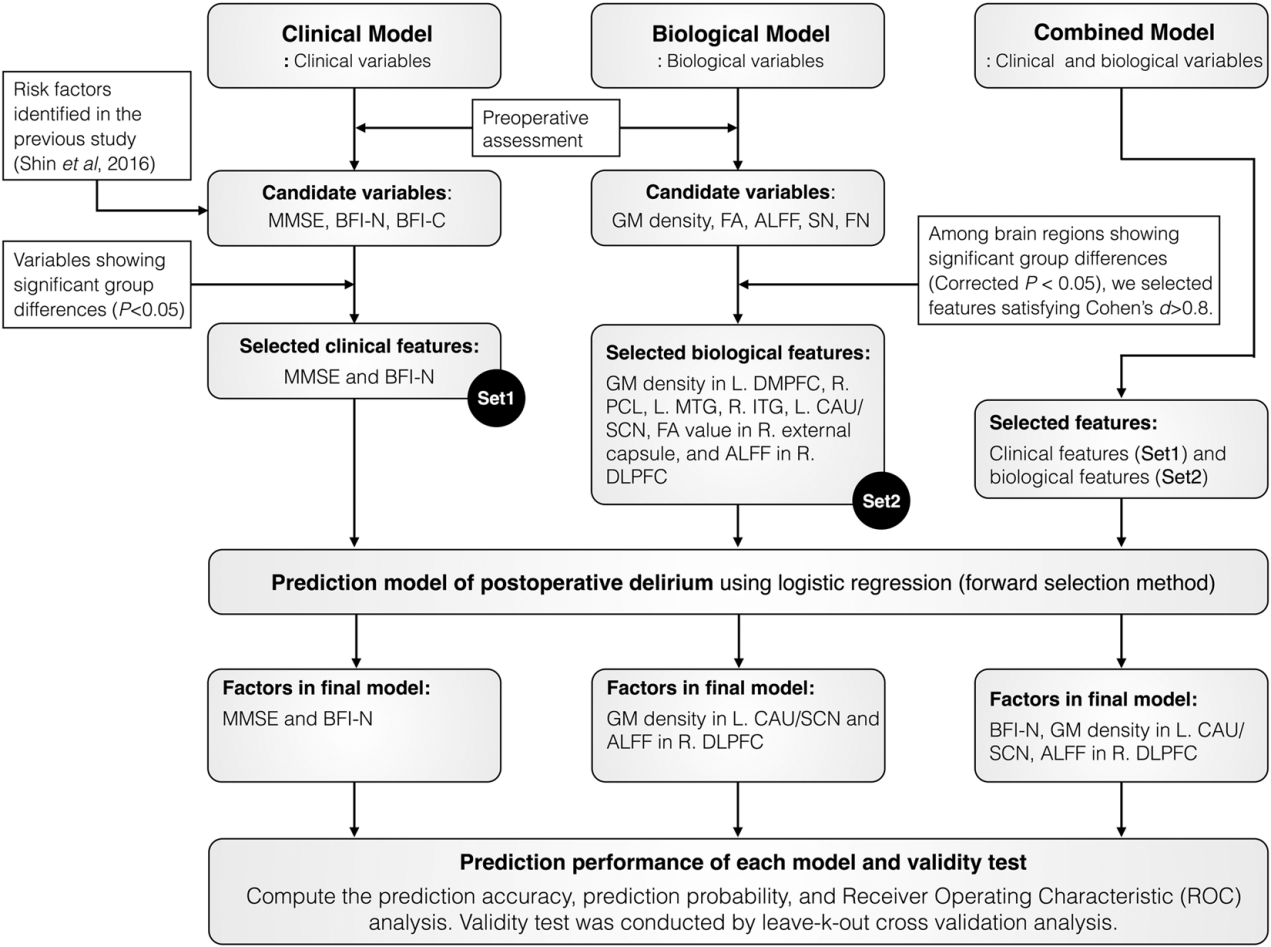
Flowchart of the prediction model for postoperative delirium from variable selection to validity test. Abbreviations: ALFF; amplitude of low-frequency fluctuation; BFI, Big Five Inventory; BFI-C, conscientiousness of the BFI; BFI-N, neuroticism of the BFI; CAU, caudate; DLPFC, dorsolateral prefrontal cortex; DMPFC, dorsomedial prefrontal cortex; FA, factional anisotropy; FN, functional network; GM, gray matter; ITG, inferior temporal gyrus; MMSE, Mini-Mental State Examination; MTG, middle temporal gyrus; PCL, paracentral lobule; SCN, suprachiasmatic nucleus; and SN, structural network.

Fig. 4 shows the flowchart of the prediction model for postoperative delirium from variable selection to validation, and presents the final model obtained from logistic regression after applying the forward selection method. Input features for the clinical model included the MMSE score and neuroticism score from the BFI since these variables showed a significant group difference. GM density, FA, ALFF, and small-world network properties were input features for the biological model. Among all preoperative biological variables showing significant differences between the two groups of participants who developed and did not develop postoperative delirium, biological measures with a large effect size (Cohen’s *d* >0.8) were selected as input variables for the biological model. In principle, the prediction model showed better performance when distinguishable features between the groups were entered in the model, therefore a large effect size was helpful for discriminating between the groups.

As illustrated in Fig. 4, we tested three prediction models such as the clinical, biological, and combined models. In the clinical model with the medical and psychological factors as input variables, variables that remained significant in the forward selection method were the MMSE score (*β* = −0.11; OR, 0.90; 95% confidence interval (CI), 0.80–1.00; *P* = 0.05) and neuroticism score (*β* = 1.01; OR, 2.75; 95% CI, 1.51–5.02; *P* < 0.01). The biological factors showing significant group differences with a large effect size (Cohen’s *d* > 0.8) were included in the biological model as well as in the combined model. In the biological model with various neuroimaging features as input variables, significant factors were GM density in the left caudate/suprachiasmatic nucleus (*β* = −23.97; OR, 0.00; 95% CI, 0.00–0.001; *P* < 0.01) and ALFF in the right DLPFC (*β* = 3.63; OR, 37.72; 95% CI, 4.38–324.90; *P* < 0.01). Finally, in the combined model with all clinical and neuroimaging factors as input features, variables that remained significant in the combined model were the neuroticism score (*β* = 1.26; OR, 3.51; 95% CI, 1.48–8.31; *P* < 0.01), GM density in the left caudate/suprachiasmatic nucleus (*β* = −24.15; OR, 0.00; 95% CI, 0.00–0.006; *P* = 0.01), and ALFF in the right DLPFC (*β* = 3.80; OR, 44.59; 95% CI, 3.61–550.76; *P* < 0.01). All three models were well fitted with *P* > 0.3 in the Hosmer and Lemeshow goodness-of-fit test.

### Classification and Predicted Probability

**Figure 5:**
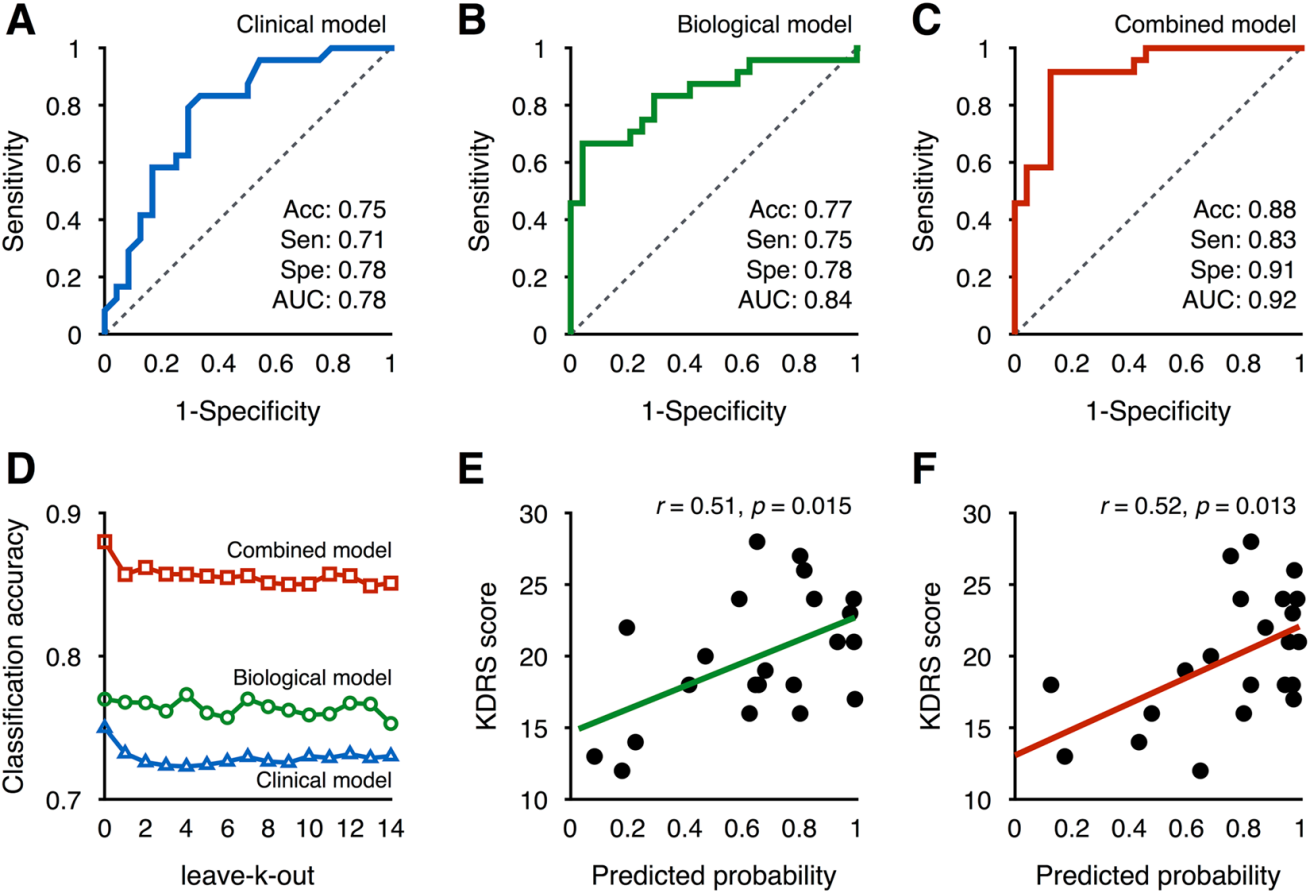
Performance of the delirium prediction models and correlations between the predicted probability and severity of postoperative delirium: Analysis of the receiver operating characteristics for the clinical (**A**), biological (**B**), and combined (**C**) model; leave-*k*-out cross validation analysis for each model (**D**); and Pearson’s correlation analysis between the Korean version of Delirium Rating Scale (KDRS) score and the predicted probability of developing postoperative delirium obtained from the biological model (**E**) and combined model (**F**), respectively.

Fig. 5A–C shows the receiver operating characteristic curves evaluating each model’s classification performance. For the clinical model, classification accuracy (0.75), sensitivity (0.71), specificity (0.78), and area under the receiver operating characteristic curve (AUC) (0.83) were obtained using the MMSE and neuroticism scores. Here, accuracy indicates the ratio of correct identification of both delirium and non-delirium. In contrast, classification accuracy (0.77), sensitivity (0.75), specificity (0.78), and AUC (0.84) were obtained for the biological model using GM density in the caudate/suprachiasmatic nucleus and ALFF in the DLPFC. Lastly, we confirmed that the classification performances for the combined model were increased using the neuroticism score, GM density in the caudate/suprachiasmatic nucleus, and ALFF in the DLPFC: accuracy (0.88), sensitivity (0.83), specificity (0.91), and AUC (0.92). These classification performances were validated with leave-*k*-out cross-validation approaches (Fig. 5D). For all models, classification accuracy fluctuated slightly across different *k* values. However, leave-*k*-out cross-validation revealed that our models showed robust performances in classification accuracy: clinical, 0.72–0.73; biological, 0.75–0.77; and combined, 0.85–0.86. Meanwhile, as shown in Fig. 5E,F, the severity of delirium was significantly correlated with the predicted probability obtained from the biological model (*r* = 0.51, *P* = 0.015) and combined models (*r* = 0.52, *P* = 0.013), but not from the clinical model (*r* = 0.22, *P* = 0.324).

## Discussion

Based on preoperative assessments, we found that GM density in the caudate/suprachiasmatic nucleus, ALFF in the DLPFC, and neuroticism scores were the predisposing factors contributing to postoperative delirium. We further developed a prediction model, and extended prediction studies to estimate the severity of delirium. The coefficients obtained from logistic regression were used to compute a predicted probability. Interestingly, this probability was significantly associated with the severity of delirium. Considering that both structural and functional information are involved in the prediction of postoperative delirium, individual differences in structural atrophy and functional abnormalities may be important in predicting individual variations in the severity of delirium.

The current prediction models confirm the previous finding that neurotic personality contributed to the development of postoperative delirium^5^. It is worth noting that the importance of neuroticism was repeatedly observed in the clinical and combined models with a relatively small sample size compared to our previous study. However, conscientiousness in personality traits, which was significant in the previous study, was not included in the present model because this factor showed no significant difference between the DEL and No-DEL groups. Considering that higher neuroticism has been related to increased stress responses^16,17^ and elevated levels of inflammatory cytokine^18^, neurotic personality might contribute to the development of postoperative delirium.

Meanwhile, the MMSE scores were significantly lower in the DEL group than in the No-DEL group. In many studies, cognitive impairment was one of the most frequently reported risk factors of postoperative delirium^19,20^. Given that a decline in cognitive function is a major symptom of dementia^21,22^, it is not surprising that dementia and delirium are mutually associated. Cognitive impairment in dementia has been associated with alterations of the structure^23^ and function^24,25^ of the brain, which in turn can be a neural predisposing factor of delirium. In order to leave a common neural factor, we did not include the MMSE score as a covariate in group-level neuroimaging data analysis. For the same reason, however, our analysis has the limitation that it cannot rule out the possibility that the resultant findings are due to dementia itself.

Relative to the No-DEL group, the DEL group showed significant increase of the characteristic path length computed from the preoperative structural network, but not from the functional networks. In principle, the characteristic path length of the structural network represents the efficiency of the physical network backbone^26^, whereas that of the functional network indicates functional integration^27^ and intellectual performance^27^. Therefore, it seems that the physical pathway was inefficient in the DEL group, but the ability to integrate functional information in the DEL group was similar to the No-DEL group due to neural plasticity^28^ and functional network reorganization^29^. Considering that an increased characteristic path length computed from functional connectivity has been observed in dementia^30,31^ and the topological organization of the functional network is closely related to resilience against attack^32^, our result suggested that functional network organization in the DEL group was more recovered from pathological state like an acute confusion state to original state than dementia.

Moving our focus on to structural abnormalities, voxel-wise statistical comparisons identified the structural predisposing components. For instance, relative to the No-DEL group, the DEL group showed decreased GM density in multiple temporal regions. In particular, GM density in the middle temporal gyrus and hippocampus was significantly correlated with the severity of perceptual disturbances and hallucinations. Indeed, visual hallucinations have been related to abnormality in the temporal lobe^33^. In particular, the temporal cortex is involved in the integration of visual perception and memory^34^. Therefore, structural atrophy of the temporal regions in the DEL group might reflect the vulnerability to perceptual disturbances and hallucinations during an episode of delirium.

Meanwhile, relative to the No-DEL group, the DEL group showed increased ALFF in the DLPFC and decreased GM density in the default mode network such as the dorsomedial prefrontal cortex and PCC. Anti-correlation between DLPFC and PCC activities, which is an intrinsic property of functional connectivity as they are the task-positive and task-negative relationships, respectively, has been observed to be lost during an episode of delirium^11^. Given that a major function of the DLPFC and PCC has been associated with executive control^35^ and consciousness^36^, respectively, our results suggest that functional hyper-fluctuations in the executive control area and the structural disruption in the default mode network might be a source of flipping the polarity of functional connectivity when patients are experiencing delirium. In particular, since the DLPFC plays a pivotal role in executive control^37^, increased ALFF in this region may be associated with proneness to the abnormal cognitive process in delirium. Previous studies have demonstrated that prefrontal ALFF changes may be related to impaired thought processes or psychotic behaviors^38,39^. Taken together, the ALFF can be effective in identifying the neural predisposing factors of delirium and estimating the probability of postoperative delirium.

Another predisposing factor may be the subcortical components. For example, the DEL group showed abnormal GM density in the caudate nucleus compared with the No-DEL group. The FA value in the external capsule, which is located close to the putamen, was significantly decreased in the DEL group and was significantly associated with the severity of delirium. The caudate and putamen are the main components of the striatum and belong to the frontostriatal connectivity loop by receiving information from prefrontal regions including the DLPFC^40^. Given that delirious patients show an inattentive behavior and impaired cognition, striatal structural abnormalities could be related to functional disconnection in the frontostriatal connectivity loop and this subcortical atrophy might be a source of attention loss when patients are in delirium.

The last considering factor is GM density in the bilateral suprachiasmatic nucleus, which has been known as the master pacemaker driving 24-hour rhythms in both physiology and behavior^41^, Indeed, disrupted sleep-wake cycles and diurnal variations in symptom severity are very common in delirium and have been proposed as core criteria for the diagnosis^42^. Taken together, our results suggest that structural atrophy in this nucleus might be related to vulnerability to disrupted physiological and behavioral circadian rhythms in delirium.

Our prediction model provides some clues to future studies on personalized clinical administration for preventing postoperative delirium. Considering that postoperative delirium can occur in all surgical patients in various clinical divisions and occurrence of delirium has adverse effects on the prognosis of postoperative patients^20^, the development of an effective prevention model may be a good starting point toward accurate and early intervention for postoperative delirium. Our prediction model has realistic aspects for clinical application in that the computation time for estimating the probability of postoperative delirium for a new patient takes less than 2 hours including assessments of neuroticism score and MRI scanning before performing surgery. Doctors might consider general anesthesia for vulnerable orthopedic patients to decrease stress by hearing the sound of a hammer and drill during surgery under regional anesthesia^5^. Considering that postoperative pain management strategies contribute to the incidence of postoperative delirium in geriatric patients, flexible administration of a patient-specific sedative and analgesic medications for vulnerable patients may be important^43^. It is likely, however, that the clinical value and cost-effectiveness would be low in the current practice for elderly patients with femoral neck fracture as personality tests and expensive MRI scans are not directly related to the fracture and operation and thus are not being performed on orthopedic inpatients.

This study has some additional limitations. First, the generalization of our prediction model for postoperative delirium might be limited because we only recruited patients with femoral neck fracture. Second, as suggested in the introduction, we included only the MMSE and BFI scores as a preoperative clinical variable, based on our previous findings^5^. Because the development of delirium may be multifactorial, we cannot exclude the effect of other unknown clinical factors that were not assessed in the study. Third, we could not extend our prediction model to identify subtypes of delirium due to a limited sample size. Forth, although levels of consciousness vary depending on the time of the day, we did not control the acquisition time of imaging data because patients who fractured their femoral neck were not always cooperative.

In summary, based on the preoperative psychological and neuroimaging assessments, we created the prediction models to estimate an individual’s probability of developing postoperative delirium using a logistic regression approach. We found that the neuroticism score, ALFF in the DLPFC and GM density in the caudate/suprachiasmatic nucleus were predisposing factors. Given that these factors are closely related to symptoms of delirium such as aberrant stress responses, impaired cognitive control, and perceptual disturbances, our prediction model may estimate the probability of postoperative delirium. In light of our results, clinical studies on a “delirium prevention model” can hopefully open doors to new treatment innovations that may reduce the incidence of postoperative delirium.

## Material and Methods

### Participants and Assessment of Delirium

Demographic and clinical information was collected before the surgery for femoral neck fracture. Participants’ cognitive level was measured using the MMSE^44^. Participants’ personality was assessed using the short form of the BFI^45^. After the surgery, a diagnosis of delirium was made according to DSM-IV criteria based on clinical interviews with a trained psychiatrist. Follow-up assessments were performed every day until the 5th day after the surgery if postoperative delirium was not diagnosed. The severity of delirium was assessed using the KDRS^46^. We obtained written informed consent from the participants or their surrogates after giving them a complete description of the study. This study was approved by the institutional review board of Yonsei University Gangnam Severance Hospital and carried out in accordance with the Declaration of Helsinki.

### Image Acquisition and Computation of Neuroimaging Measures

Preoperative neuroimaging data including the high resolution T1-weighted imaging, diffusion tensor imaging (DTI), and rsfMRI were obtained from all participants one day before the surgery with a Sigma EXITE 3.0 Tesla MR system (GE, Milwaukee). A T1-weighted anatomical image was obtained using a spoiled gradient-echo sequence (matrix = 256 × 256, echo time = 3.2 ms, repetition time = 8.2 ms, field of view = 240 mm, slice thickness = 1.2 mm, flip angle = 12°, and number of slices = 136) to serve as an anatomical underlay for the brain activity and to be used for GM volume analysis. DTI data were acquired using a single shot spin-echo planar imaging sequence (matrix = 128 × 128, repetition time = 8,000 ms, field of view = 240 mm, and slice thickness = 2.6 mm, number of slices = 64). Sixteen DTI volumes were obtained for each participant, including 15 volumes with diffusion gradients applied along 15 non-collinear directions (b = 1000 s/mm^2^) and one volume without diffusion weighting (b = 0). Resting state functional images were obtained using over 5 minutes using gradient-echo echo-planar imaging sequences (matrix = 64 × 64, echo time = 17.6 ms, repetition time = 2,000 ms, field of view = 240 mm, slice thickness = 3 mm, flip angle = 90°, number of slices = 50). All participants were instructed to rest with their eyes closed during the scan. Due to an unexpected error during data acquisition, DTI and rsfMRI from one patient with delirium were excluded in all imaging analysis.

### Preprocessing of DTI

Visual inspection of all diffusion-weighted images was conducted. For each subject, diffusion-weighted images were registered to the corresponding b = 0 image with an affine transformation to correct distortions due to eddy current (FSL 5.0.9; http://www.fmrib.ox.ac.uk/fsl). FA maps were constructed in native space using the Diffusion Toolkit. These FA maps were then further processed for the second-level statistical analysis as described below (see voxel-based morphometry section). The white matter tracts of the brain structural networks were reconstructed by using the deterministic fiber tracking method, based on a fiber assignment by continuous tracking (FACT) algorithm^47^. Within each voxel in the brain mask, one seed was started, evenly distributed over the volume of the voxel. A streamline was started from each seed following the principal diffusion direction from voxel to voxel, thus reconstructing white matter fibers. Stopping criteria were used as following: an angular threshold of 60°, FA threshold of 0.1, and the track length of 5 mm.

### Voxel-wise analyses of GM and FA

Voxel-wise analyses of GM density and FA were conducted using DARTEL toolbox implemented in SPM12 software^48^. Individual anatomical and FA images were visually inspected for any artifacts or anatomical abnormalities. Before conducting voxel-wise statistical analysis, FA maps were coregistered to individual anatomical images. Then, T1-weighted MR images were segmented into GM, white matter and cerebrospinal fluid provided by SPM12 default segmentation model. A study-specific GM template was generated based on the scans of all participants. The segmented GM images and coregistered FA images were nonlinearly normalized to the study-specific templates. Spatially normalized images were then modulated using the Jacobian determinant of the deformation field to adjust for volume changes during nonlinear transformation^49^. These modulated GM and FA maps were smoothed using a 6-mm full-width half-maximum isotropic Gaussian kernel.

### Preprocessing of rsMRI Data

Preprocessing of rsfMRI data was conducted using SPM12 software. The time series data for the first ten seconds were discarded to eliminate any signal decay associated with the magnetization reaching equilibrium. After realignment for head motion correction, the corrected images were coregistered to the T1-weighted image for each subject. The T1-weighted image and all rsfMRI data were then normalized to the Montreal Neurological Institute (MNI) template space. Then, the normalized functional images were smoothed using a Gaussian filter with a 6-mm full-width at half-maximum. To remove confounding effects, we regressed out artifacts from head motions and physiological noises from the white matter and cerebrospinal fluid. After regressing out all nuisance parameters, residual time-series data were used for evaluating the ALFF^50^, which was computed as local metric to capture alterations in the resting state blood-oxygenation-level-dependent signal.

### Network construction and small-world network measures

To proceed the structural and functional network construction, cortical and sub-cortical brain areas were parcellated into 90 regions of interests (ROIs) using the automated anatomical labeling (AAL) atlas^51^. A total of 90 anatomically defined ROIs covered the whole-brain except for the cerebellum. Although several studies ignored subcortical parts of the brain in the network construction^52,53^, we included these regions such as the thalamus, caudate, putamen, and pallidum because these subcortical structures have kept vital connections with cortical areas^54–56^.

The structural networks were constructed through the following steps. To obtain the transformation matrix from the MNI to individual native space, we conducted coregistration of the T1-weighted image to b = 0 image, and then normalization of the coregistered T1-weighted image to the standard MNI template image. The non-linear inverse transformation matrix was applied to the AAL parcellation atlas such to generate corresponding parcellation volumes in each individual’s native space of the diffusion-weighted image. Then, individually fitted parcellation map divided the whole-brain into 90 cortical regions in the individual space. Finally, we constructed the weighted structural network using FA value. To assign connection weight between two regions, the voxel-wise FA values for the existing streamlines were extracted and averaged for each edge connecting region i and j. Edges having fewer than three streamlines were considered potentially spurious and were deleted from the connection matrix.

The mean time series within each ROI were obtained from the cortical parcellation method. The adjacency matrix (Aij) for each subject was computed using Pearson’s correlation coefficients between the i-th and j-th mean time-series for each parcellation, respectively. We then obtained the sparse functional network by applying a threshold of Bonferroni corrected P < 0.05 on the functional connectivity strength.

We then computed global metric such as small-world network properties, including global efficiency, characteristic path length, local efficiency, and average clustering coefficients. These measures were normalized using that of 10,000 random networks^26^.

### Statistical analysis

To identify preoperative neural predisposing factors of delirium, second-level random effect analysis was conducted using SPM12 software to assess any differences in GM, FA, and ALFF between the DEL and No-DEL groups. All reported regional clusters survived at a corrected *P* < 0.05 threshold which corresponds to the family-wise error corrected significance at the cluster level with a cluster-defining threshold of *P* < 0.001. Total intracranial volume was included as a confounding regressor during an evaluation of group differences in GM and FA values. To determine significant group differences in network parameters, we applied independent sample *t*-test with a statistical threshold of *P* < 0.05. Meanwhile, in order to exclude a bias during the acquisition of the functional data, the framewise displacement representing head movements^57^ during rsfMRI scans was compared between the two groups. Finally, partial correlation analysis was conducted to determine relationships between the neural predisposing factors and KDRS severity scores while controlling for total intracranial volume. Particularly, relationships between the regional ALFF and KDRS severity scores were evaluated with Pearson’s correlations and statistically significant correlations were obtained at a threshold of corrected *P* < 0.05, correcting for multiple comparisons using the Benjamini-Hochberg procedure^58^.

Furthermore, the logistic regression approach was chosen for prediction analysis because the predicted probability of a disease produced by the logistic regression is a continuous variable and has an advantage for further analysis such as a correlation with the symptom severity. We conducted logistic regression analyses using the Statistical Package for the Social Sciences (SPSS) Version 22.0 (IBM Corp., NY, USA) with three different prediction models: clinical model, biological model, and combined models. For each model using clinical, biological, and combined datasets, the final factors that remained in the prediction models were determined by the forward selection method of logistic regression.

For each prediction model, we computed accuracy, sensitivity, specificity, and AUC. Based on the parameter estimation, we obtained the probability for postoperative delirium. We then computed the correlation coefficients between delirium probability and symptom severity. To test the validity, we performed leave-*k*-out cross-validation methods where *k* ran from 1 to 14. For example, *k* = 1 (*k* = 14) indicated that the parameter estimation was done by using *N* = 55 (*N* = 42) of 56 and predicted the remaining 1 (14) sample(s).

The predicted probability was computed using the regression coefficients obtained from logistic regression. Once we obtained the regression coefficients (*β*_*i*_), the odds ratio (OR) could be evaluated as ${\rm{odds}}=\exp ({\beta }_{0}+{\beta }_{1}{X}_{1}+{\beta }_{2}{X}_{2}).$ Then, the individually predicted probability could be computed as odds/(1+odds). In case of the combined model, the number of regression coefficients increased to 4 including *β*_0_.
